# Sarcopenia in Non-Dialysis Chronic Kidney Disease Patients: Prevalence and Associated Factors

**DOI:** 10.3389/fmed.2022.854410

**Published:** 2022-04-07

**Authors:** Geraldo José de Amorim, Cinthia Katiane Martins Calado, Bruno Carlos Souza de Oliveira, Renata Patricia Oliveira Araujo, Tayrine Ordonio Filgueira, Matheus Santos de Sousa Fernandes, Angela Castoldi, Gisele Vajgel, Lucila Maria Valente, José Luiz de Lima-Filho, Paulo Roberto Cavalcanti Carvalho, Fabricio Oliveira Souto

**Affiliations:** ^1^Nephrology Service, Hospital das Clínicas, Federal University of Pernambuco, Recife, Brazil; ^2^Laboratory of Immunopathology Keizo Asami (LIKA/Federal University of Pernambuco (UFPE)), Recife, Brazil

**Keywords:** chronic kidney disease, sarcopenia, bioelectrical impedance, renin-angiotensin-aldosterone system, phase angle, systemic chronic inflammation

## Abstract

**Background:**

Sarcopenia is related to morbidity and mortality in non-dialysis Chronic Kidney Disease (ND-CKD) patients; however, the pathophysiology of sarcopenia remains unclear. The study aimed to assess the prevalence and factors associated with sarcopenia in ND-CKD individuals.

**Methods:**

We cross-sectionally evaluated 139 prevalent ND-CKD patients attending our outpatient clinic at Hospital das Clínicas of the Federal University of Pernambuco, between April and October 2019. Patients older than 18 years old and at G3-G5 CKD stages were included. Hand grip strength, Muscle Mass appendicular Index, and Gait Speed (GS) were defined by the standards of the European Working Group on Sarcopenia in Older People 2 guideline.

**Results:**

Sarcopenia prevalence was 20.9% and severe sarcopenia 2.9%. Sarcopenic were mostly found in elderly ones (64.8 ± 13.5 years vs. 54.9 ± 12.8 years, *p* < 0.001), revealing lower body mass index [26.1 (6.8) vs. 28.6 (6.2), *p* = 0.023], lower phase angle (PhA) [4.50 (1.10) vs. 5.60 (1.20), *p* < 0.001] and lower GS [1.00 (0.50) vs. 1.40 (0.4), *p* < 0.001]. They also presented lower serum creatinine levels [2.40 (1.50) vs. 3.0 (1.8), *p* = 0.032], lower Albumin-to-Creatinine Ratio [72.60 (1008.30) vs. 342.30 (1172.1), *p* = 0.039] and Hemoglobin levels [11.45 (1.8) vs. 12.60 (2.40), *p* = 0.003], and higher levels of C-reactive protein [0.2 (0.80) vs. 0.03 (0.3), *p* = 0.045] compared to non-sarcopenic. Under Poisson Multivariate Model, PhA [Relative precision (RP): 0.364, Confidence Interval (CI) (95%):0.259–0.511, *p* < 0.001], Interleukin six (IL-6) [RP: 1.006, CI (95%):1.001–1.01, *p* = 0.02] and serum creatinine levels [RP: 0.788, CI (95%): 0.641–0.969, *p* = 0.024] were associated with sarcopenia.

**Conclusions:**

Sarcopenia predominance was identified in our ND-CKD population, and was associated with lower PhA values, higher IL-6 levels, and lower serum creatinine levels.

## Introduction

Chronic kidney disease (CKD) is a prevalent condition, globally estimated in 14.3% of the world's population, and in 36.1% of the population at risk, such as those living in the Middle East, Southeast Asia, and Eastern Europe (1). Sarcopenia, defined as a loss of muscle mass, quality, and function, is related to an increased mortality and morbidity, frailty, and hospitalizations in the CKD population. Sarcopenia prevalence in Non-Dialysis CKD patients (ND-CKD) varies between 5.9 and 28.7%, according to the outcomes of the European Guideline Working Group on Sarcopenia in Older People (EWGSOP2) is applied (2–4).

Uremic Sarcopenia has multifactorial pathophysiology, including a systemic chronic inflammation, a decrease of anabolic hormones like type 1 Insulin Growth Factor (IGF-1), testosterone and active vitamin D, an increase of myostatin and Angiotensin 2 (AT2) levels, as well as metabolic acidosis occurrence (5); besides sedentarism and malnutrition (6, 7). Pathophysiological mechanisms through which elevation of inflammatory markers promotes changes in muscle mass are probably connected to an imbalance in muscle protein turnover, with activation of the ubiquitin-proteasome system, stimulation of cell death, and apoptosis, which compromises the muscle regeneration capacity (8). Although it is known that systemic chronic inflammation and increased inflammatory markers such as C-reactive protein (CRP), Interleukin six (IL-6), and Tumor Necrosis Factor-alpha (TNF-α), are presented by ND-CKD patients and yet could increase muscle mass degradation and sarcopenia in this population (5, 9) these findings are still controversial in some researches (3, 7).

An outgrowth in serum AT2 levels and the role of the Renin-Angiotensin-Aldosterone System (RAAS) in uremic sarcopenia pathophysiology have been described in recent studies (10, 11). The benefits of Angiotensin-Converting Enzyme Inhibitors (ACEI) or Angiotensin II Receptor Blockers (ARBs) in CKD patients are related to its anti-hypertensive, antiproteinuric, anti-inflammatory, and immunomodulatory effects (12). However, the role of ARBs-blockers for sarcopenia prevention or management in the ND-CKD population is indeed unknown.

Phase Angle (PhA) is a parameter derived from Bioelectrical Impedance Analysis (BIA) (13) and it has been shown as a good predictor of muscle mass and function in many populations (14, 15). Whilst, EWGSOP2 determined that PhA can be used as a marker of muscle quality in sarcopenia assessment (16) and that, in ND-CKD patients, PhA was related to nutritional parameters (17), its role as a predictor of sarcopenia in this population is undisclosed.

While knowledge about sarcopenia pathophysiology has improved, the diagnosis and the treatment of this condition in ND-CKD patients represent a challenge for nephrologist's daily clinical practice. In addition, uremic sarcopenia research in ND-CKD patients is still scarce and the role of ARBs-blockers, PhA derived BIA, and inflammatory markers as predictors of sarcopenia in this population have not been well-established. The purpose of our study is to determine sarcopenia prevalence along with the role of PhA, ARBs-blockers, and inflammatory markers occurring in ND-CKD patients.

## Methods

### Study Design

This study was a prospective clinical trial. We cross-sectionally evaluated 139 prevalent ND-CKD patients attending our outpatient clinic at Hospital das Clínicas of the Federal University of Pernambuco (HC-UFPE), between April and October 2019. Our study was approved by the Research Ethics Committee of HC-UFPE, number 3.258.943. All individuals gave their prior consent before placing any study.

### Sample Size Calculation

The calculation of sample size was determined by the following equation:


(1) $$n=\frac{z^{2}.\;p\;.\;q.\;N}{d^{2}\;.\left(N-1\right)+z^{2}\;.\;p\;.\;q}$$

Where:

*z* = quantile of the standard normal distribution (1.96 at 95% confidence);

*p* = expected prevalence of patients with sarcopenia (*p* = 0.2);

*q* = expected prevalence of patients without sarcopenia (*p* = 1–*p* = 1–0.2 = 0.8);

*d* = sampling error (*d* = 0.05);

*N* = Total number of patients treated at the service (*N* = 350).

Considering the expected prevalence of 18% for the number of patients with sarcopenia and the total number of 350 patients treated at the CKD outpatient clinic, we have that the number of individuals required to sample is 138. The collection was carried out intentionally, in which all patients who met the inclusion criteria were evaluated until completing the minimum sample size for the study.

### Inclusion and Exclusion Criteria

**Figure 1:**
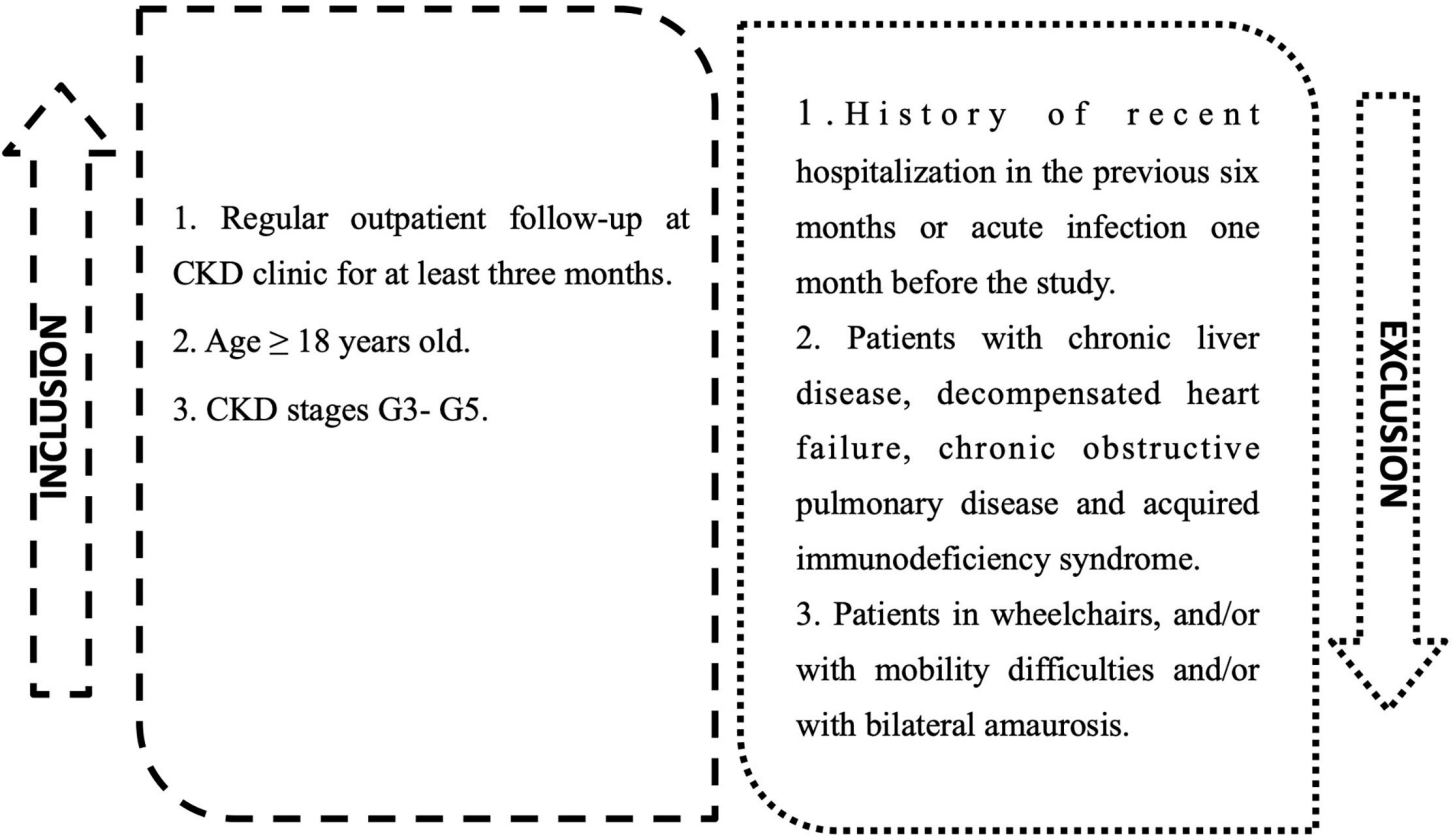
Inclusions and exclusions criteria for study group.

Inclusion criteria: (1) Regular Attending in CKD Outpatient clinic for at least 3 months; (2) Age ≥18 years; (3) Classified as CKD stages G3-G5. We excluded patients with a history of recent hospitalization and patients with chronic liver disease, decompensated heart failure, chronic obstructive pulmonary disease, and acquired immunodeficiency syndrome; wheelchair patients, and/or with limited mobility and/or bilateral amaurosis (Figure 1).

### CKD Stage and Clinical Data

CKD stage was defined according to kidney disease: Improving Global Outcomes criteria (18), and estimated Glomerular Filtration Rate (eGFR), was calculated using Chronic Kidney equation Disease Epidemiology Collaboration (19). Sociodemographic information and etiological causes of CKD, such as, systemic arterial hypertension (SAH); diabetes mellitus (DM); chronic tubulointerstitial nephritis; chronic glomerulonephritis, and autosomal dominant polycystic kidney disease, were collected from medical records.

### Laboratorial and Drugs Data Collection

Laboratory data were collected from medical records including serum hemoglobin, urea, creatinine, intact parathyroid hormone, alkaline phosphatase, calcium, phosphorus, albumin, magnesium, vitamin D, ferritin, total testosterone, alkaline reserve, the albumin-to-creatinine ratio in a urine sample (ACR), C-reactive protein (CRP). Drugs evaluated were ARBs, ACEI, and furosemide used for a period equal to or >3 months.

### Sarcopenia Diagnosis

Sarcopenia diagnosis was assessed according to EWGSOP2 (16) guidelines. Handgrip Strength (*HGS*) was applied to evaluate muscle strength, and muscle mass was determined by appendicular muscle mass index (APMMI/m^2^). To evaluate patients' muscle performance Gait Speed (*GS*) was performed.

### Anthropometric and Bioelectrical Impedance Data

Body Weight and height were measured at a weighing and measuring station (GmbH & Co. KG, Hamburg, Germany), in order to calculate Body Mass Index (BMI). Additionally, waist circumference (WC) was measured at midpoints between the last rib and upper border of the iliac crest. Body composition was assessed using an octopolar BIA device (seca mBCA 525; seca gmbh & co. Kg, Hamburg, Germany) that uses a 4-body compartment model (20). Data obtained were evaluated by Seca analytics 115 software, providing parameters of skeletal muscle mass (SMM), appendicular skeletal muscle mass (APMM), and PhA (Equation 1). From APMM data, the APMMI/m^2^ was calculated according to Equation (2). Values <7.0 kg/m^2^ in men, and <5.5 kg/m^2^ in women were considered altered (16).


(2) $$Phase\;Angle\;(PhA)=\;\frac{Reactance\;(Xc)}{Resistance\;(R)}\times \;\left(\frac{180}{\pi }\right)$$

(3) $$APMMI\;\left(kg.m^{-2}\right)\;=\;\frac{APMM}{H^{2}}$$

Where APMM is the appendicular skeletal muscle mass in kg; and H^2^ is square height in meters.

### Muscle Performance and Muscle Strength Tests

Handgrip Strength (HGS) was performed using a dynamometer (Baseline^®^, NexGen Ergonomics, Inc., Quebec, Canada) to assess muscle strength. During execution, patient was instructed to hold the dynamometer with the dominant hand in order to form a 90° angle between the arm and forearm. After receiving a verbal order from the examiner, he should print maximum force on the instrument. The reference values used were those determined by Dodds et al. and the maximum strength measures <16 kg in women and <27 kg in men were considered altered (16, 21).

*GS* was applied to assess patients' muscle performance. After this initial observation, the patient was asked to walk as quickly as possible, but without running, and the walking time, in seconds, between the second and eight meters was measured. For the quantification of time, a digital stopwatch (CASIO HS-3V-1; CASIO, São Paulo, Brazil) was used. The first two and last two meters, considered points of acceleration and deceleration of gait, were not included in the assessment. Finally, *GS* was calculated, in meters per second (m/s), dividing the distance of six meters traveled by the time measured in seconds. Values <0.8 m/s were considered altered (16).

### Plasma Cytokine Analysis

Cytokine analysis was performed in the collected blood samples to measure plasma levels of pro and anti-inflammatory cytokines: IL-2, IL-4, IL-6, IL-10, Interferon gamma (IFN-γ), and TNF-α using a CBA kit (Cytometric Bead Array, Flex, # 560484, BD Biosciences), according to the manufacturer's protocol.

### Statistical Analysis

Data analysis was performed in Statistical Package for the Social Sciences version 18 (SPSS—IBM Corporation, New York, NY, USA). The profile distribution between the CKD classification groups and their homogeneity was analyzed using the Chi-square test. Then, we performed a descriptive analysis of data expressing the mean and standard deviation or median and interquartile range, depending on the standard of normality that was assessed by the Shapiro-Wilk test. Student's *t*-test and ANOVA tests were applied to compare the means between two and three or more groups, respectively. However, Mann-Whitney and Kruskal-Wallis tests were applied to non-parametric tests, aiming to compare two or more groups, respectively. To assess the association with sarcopenia, the Chi-square test for independence was applied and, in cases where the assumptions were violated, Fisher's exact test was applied. Variables associated to sarcopenia, with a *p*-value up to 0.20 (20%) were included in the Poisson Multivariate Model (22). Initially, the model was adjusted by age, gender, BMI, Visceral Fat, *GS*, ARBs and ACEI use, daily protein intake, IL-10 e IL-6 levels, hemoglobin, ACR, CRP, alkaline phosphatase, and alkaline reserve. Those variables that maintained a *p*-value <0.05, after adjustment, were submitted to the final multivariate model. Finally, prevalence ratio and confidence intervals were calculated to determine the association of each factor with sarcopenia. All conclusions were established considering the significance level of 5%.

## Results

In our 139 ND-CKD patients, 53.2% were female, with a mean age of 57 ± 13.5 years. SAH was the most prevalent etiology of CKD (42.4%), followed by DM (38.1%). According to the CKD stage, most patients were classified as G4 and G5 (82.0%) (Table 1). Besides, Sarcopenia prevalence was 20.9% (*n* = 29 cases) and severe sarcopenia was found in 2.9% (*n* = 4 cases). Although sarcopenia prevalence was higher in most advanced CKD stages (79.3% in G4 and G5 vs. 20.7% in G3), it was not statistically significant (Table 1).

**Table 1:** Distribution of sarcopenia according to sociodemographic, clinical and body composition characteristics in the CKD study population.

**Variables**	**Missing data**	**Total (*n* = 139)**	**Sarcopenic (*n* = 33)**	**Non-sarcopenic (*n* = 106)**	***p*** **-value**
**Age (years)**	0 (0.0%)	57.0 ± 13.5	64.8 ± 13.5	54.9 ± 12.7	<0.001^c^
**Sex**					
Female	0 (0.0%)	74 (53.2%)	20 (27.0%)	54 (73.0%)	0.056^a^
Male		65 (46.8%)	9 (13.8%)	56 (86.2%)	0.056^a^
**CKD stage** ***n*** **(%)**					
Stage 3	0 (0.0%)	25 (17.9%)	6 (20.7)	19 (17.3)	0.670^b^
Stage 4and 5		114 (82.0%)	23 (79.3%)	91 (82.7%)	
**CKD etiology**					
Systemic arterial hypertension (%)		59 (42.4%)	14 (23.7%)	45 (76.3%)	0.475^a^
Diabetes mellitus (%)		53 (38.1%)	13 (24.5%)	40 (75.5%)	0.404^a^
Chronic glomerulonephritis (%)	0 (0.0%)	24 (17.3%)	2 (8.3%)	22 (91.7%)	0.097^a^
Tubulo-interstitial-chronic nephritis (%)		19 (13.7%)	5 (26.3%)	14 (73.7%)	0.548^b^
Autosomal dominant polycystic kidney disease (%)		13 (9.4%)	1 (7.7%)	12 (92.3%)	0.301^b^
Others etiologies		10 (7.2%)	2 (20.0%)	8 (80.0%)	1.000^b^
**Anthropometric and bioelectrical impedance data**					
BMI (kg/m^2^)	0 (0.0%)	28.2 (6.4)	26.1 (6.8)	28.6 (6.2)	0.023^b^
FMI (kg/m^2^)	0 (0.0%)	10.4 ± 4.3	10.12 ± 3.9	10.5 ± 4.4	0.658^a^
Waist circumference (cm)	0 (0.0%)	94.5 ± 12.2	91.9 ± 11.4	95.2 ± 12.4	0.209^a^
Fat percentage (%)	1(0.7%)	35.3 (17.6)	38.4 (13.5)	35.3 (18.4)	0.527^b^
Visceral fat (L)	0 (0.0%)	2.8 (1.8)	2.6 (1.2)	3.0 (2.1)	0.146^b^
PhA (°)	0 (0.0%)	5.3 (1.3)	4.5 (1.1)	5.6 (1.2)	<0.001^b^

*Data are expressed as the mean ± standard deviation; median (interquartile range). CKD, Chronic Kidney Disease; BMI, Body Mass Index; FMI, Fat Mass Index; PhA, Phase Angle*.

a *p-value of the Chi-square test for independence*.

b *p-value of Fisher's exact test*.

c *p-value of the Student's t-test^2^p-value of the Mann–Whitney test*.

Sarcopenic patients were older (*p* < 0.001), had lower BMI (*p* = 0.023) and lower PhA values compared to non-sarcopenic patients (*p* < 0.001) (Table 1). Hemoglobin, ACR, and serum creatinine levels were lower in patients with sarcopenia, with *p*-values of 0,003, 0,039 and 0,032, respectively, than non-sarcopenic. Furthermore, sarcopenic individuals had higher CRP levels (*p* = 0.045). On the other hand, when pro and anti-inflammatory cytokines were analyzed, no statistically significant difference was observed in serum levels of TNF-α, IL-10, IL-6, and IL-2 between sarcopenic and non-sarcopenic ND-CKD patients (Table 2). Besides that, regarding muscle parameters, patients with sarcopenia had lower *GS* (*p* < 0.001) in bivariate analysis compared to non-sarcopenic individuals (Table 3).

**Table 2:** Distribution of sarcopenia according to biochemical parameters and inflammatory markers in the CKD study population.

**Variables**	**Missing data**	**Total (*n* = 139)**	**Sarcopenic (*n* = 33)**	**Non-sarcopenic (*n* = 106)**	**p-value^a^**
**Laboratorial data**					
Urea (mg/dL)		102.0 (49.3)	102.0 (55.0)	102.5 (48.4)	0.609
Uric Acid (mg/dL)		6.8 (2.0)	6.7 (3.0)	6.8 (2.0)	0.907
Creatinine (mg/dL)	53 (38.1%)	2.9 (1.9)	2.4 (1.5)	3.0 (1.8)	0.032
Creatinine clearance (ml/min/1.73m^2^)		20.0 (12.0)	24.0 (15.5)	19.4 (11.3)	0.212
Glucose (mg/dL)		95.0 (33.0)	93.0 (29.5)	95.7 (38.7)	0.841
Hemoglobin (g/dL)		12.2 (2.5)	11.4 (1.8)	12.6 (2.4)	0.003
Calcium (mg/dL)		9.3 (0.9)	9.2 (0.9)	9.3 (0.9)	0.732
Phosphorus (mg/dL)		4.0 (1.1)	4.2 (1.2)	4.0 (1.1)	0.201
ACR (mg/g)		262.8 (1008.2)	72.6 (1008.3)	342.3 (1172.1)	0.039
PTHi (pg/mL)	7 (5.0%)	173.0 (148.5)	162.0 (161.2)	174.0 (152.5)	0.394
Magnesium (mg/dL)	6 (4.3%)	2.3 ± 0.3	2.3 (0.4)	2.3(0.4)	0.842
Ferritin (ng/mL)	11 (7.9%)	190.0 (230.6)	161.0 (256.0)	191.0 (237.0)	0.511
Vitamin D3 (ng/mL)	13 (9.4%)	32.4 (11.9)	32.1 (12.0)	32.8 (11.9)	0.507
CRP (mg/dL)	25 (18.0%)	0.06 (0.5)	0.2 (0.8)	0.03 (0.3)	0.045
Total testosterone (ng/dL)	36 (55.4%)	506.5 ± 186.6	500.5 (183.5)	512.0 (232.8)	1.000
Albumin (g/dL)	53 (38.1%)	4.3 (0.5)	4.2 (0.6)	4.3 (0.5)	0.488
Alkaline reserve (mEq/L)	56 (40.3%)	24.0 (4.1)	26.0 (4.2)	24.0 (4.1)	0.058
Alkaline phosphatase (U/L)		219.0 (110.4)	224.5 (117.0)	218.5 (98.8)	0.155
**Pro and anti-inflammatory cytokines (pg/mL)**					
IFN-γ	3 (2.1%)	57.0 (25.1)	53.7 (36.5)	57.1 (21.7)	0.700
TNF-α	4 (2.9%)	46.9 (38.1)	47.6 (38.2)	46.6 (39.9)	0.335
IL-10	2 (1.4%)	61.7 (28.7)	67.6 (32.1)	59.4 (30.1)	0.077
IL-6	4 (2.9%)	63.9 (45.9)	70.4 (64.9)	61.1 (40.7)	0.120
IL-4	24 (17.1%)	32.8 (37.4)	29.2 (44.7)	36.2 (35.0)	0.640
IL-2	2 (1.4%)	36.4 (28.1)	38.4 (29.7)	35.6 (28.9)	0.261

*Data are expressed as the mean ± standard deviation; median (interquartile range). ACR, Albumin/Creatinine Ratio in Urine Sample; PTHi, Intact Parathormone; CRP, C-reactive protein; IFN-γ, Interferon Gamma; TNF-α, Tumor Necrosis Factor Alpha; IL-10, Interleukin 10; IL-6, Interleukin 6; IL-4, interleukin 4; IL-2, Interleukin 2*.

a *p-value of the Mann–Whitney test*.

**Table 3:** Distribution of sarcopenia according to physical activity level, physical performance tests, drug use, daily protein intake and Charlson comorbidity index in the CKD study population.

**Variables**	**Missing data**	**Total (*n* = 139)**	**Sarcopenic (*n* = 33)**	**Non-sarcopenic (*n* = 106)**	***p*** **-value**
**IPAQ questionaire *n* (%)**					
Sedentary/irregularly active	1 (0.7%)	128 (92.9%)	29 (22.7)	99 (77.3%)	0.120^a^
Active/very active		10 (7.2%)	0 (0.0)	10 (100.0)	
**GS (m/s)**					
Median (interq. amplitude)	0 (0.0%)	1.4 (0.5)	1.0 (0.5)	1.4 (0.4)	<0.001^b^
**HGS (Kg)**					
Median (interq. amplitude)	0 (0.0%)	25.0 (15.0)	15.0 (3.0)	28.0 (16.0)	<0.001^c^
**Drug use *n* (%)**					
ARBs	0 (0.0%)	43 (30.9%)	6 (14.0%)	37 (86.0%)	0.180^c^
ACEIs	0 (0.0%)	15 (10.8%)	6 (40.0%)	9 (60.0%)	0.086^a^
Furosemide	0 (0.0%)	85 (61.2%)	17 (20.0%)	68 (80.0%)	0.753^c^
**Daily protein intake *n* (%)**					
Normal or High (≥0.8 g/kg/day)	0 (0.0%)	51 (36.7%)	14 (27.5%)	37 (72.5%)	0.146^c^
Low		88 (63.3%)	15 (17.0%)	73 (83.0%)	
**CCI**					
Median (interq. amplitude)	0 (0.0%)	2.0 (3.0)	3.0 (3.0)	2.0 (2.0)	0.652^b^

*Data are expressed as the number of patients—n (%) and median (interquartile range). IPAQ, International Physical Activity Questionnaire; GS, Gait Speed; HGS, Handgrip Strength; ARBs, Angiotensin Receptor Blockers; ACEIs, Angiotensin-converting Enzyme Inhibitors; CCI, Charlson Comorbidity Index*.

a *p-value of Fisher's exact test*.

b *p-value of the Mann-Whitney test*.

c *p-value of the Chi-square test for independence*.

According to drug use (ARBs, ACEI, furosemide), physical activity level, daily protein intake, and ICC, none of these variables had a statistical association with sarcopenia in our population (Table 3).

**Figure 2:**
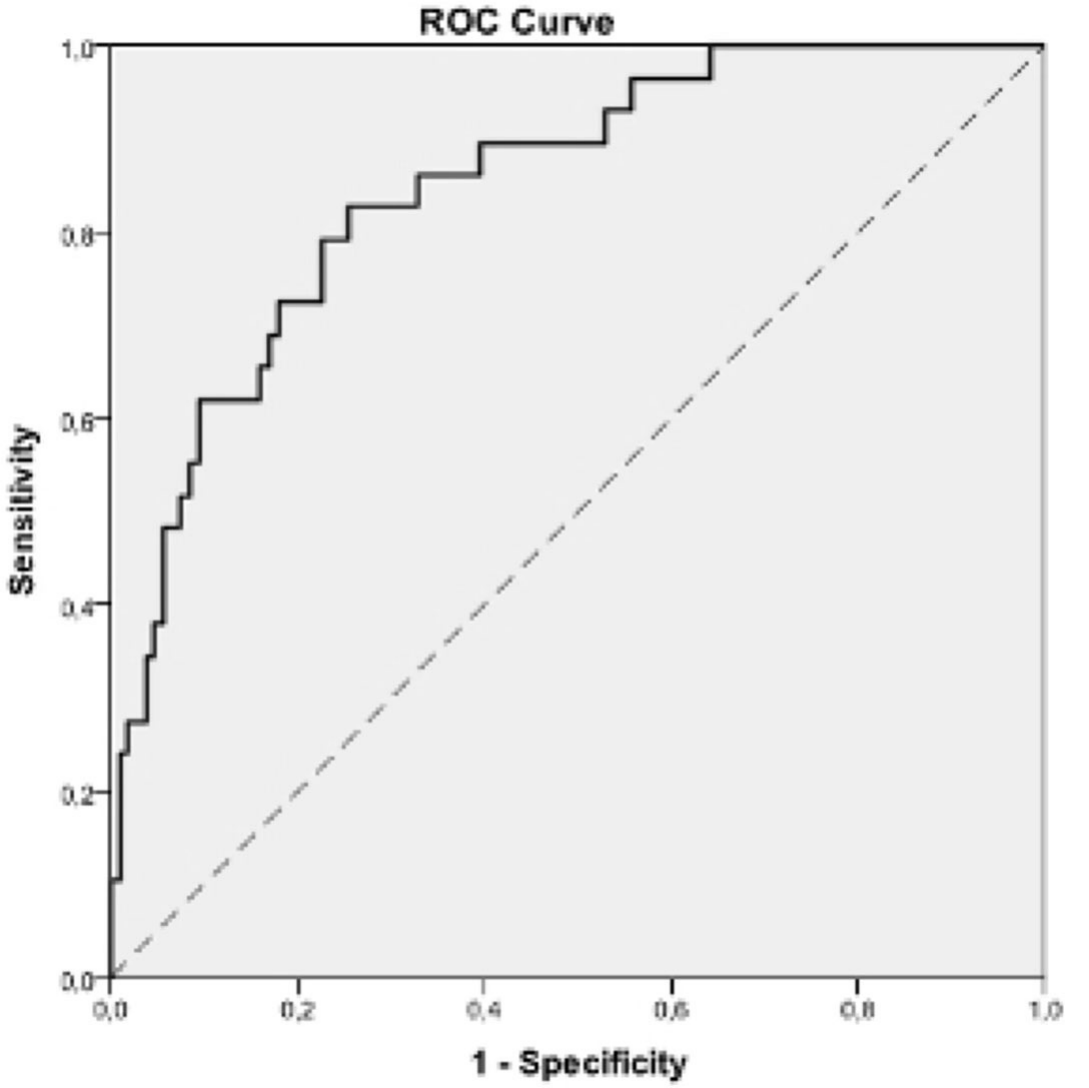
ROC analysis to predict sarcopenia outcome (Area under the curve = 0.849, *p*-value < 0.001, CI (95%) = [0.774; 0.924]). CI, Confidence Interval; ROC, receiving operating characteristic curve. Cut off value of ROC curve: 0.5.

In the Poisson Multivariate Model, the variables that remained significantly associated with sarcopenia were PhA (*p* < 0.001), IL-6 (*p* = 0.020), and serum creatinine (*p* = 0.024) (Table 4). The multivariate model demonstrates that an increase in one-unit PhA was related to 63.6% reduction [Prevalence ratio (PR) = 0.364] in the PR for sarcopenia, while an increment of 1 unit in IL-6 levels would increase the PR for sarcopenia by 0.6% (PR = 1.006). Moreover, the increase in creatinine levels would lead to a 21.2% reduction in the PR for sarcopenia. Finally, the analysis of the plotted receiver-operating characteristic curve resulted in an area under the curve of 0.849, suggesting that our model has a good association power for the outcomes evaluated (Figure 2).

**Table 4:** Multivariate poisson model derived prevalence ratio for sarcopenia by phase angle, interleukin six and creatinine.

**Factor**	**PR**	**CI (95%)**	***p*** **-value^a^**
PhA (°)	0.364	0.259–0.511	<0.001
IL-6 (pg/mL)	1.006	1.001–1.011	0.020
Creatinine (mg/dL)	0.788	0.641–0.969	0.024

*PhA, phase angle; IL-6, Interleukin 6; PR, Prevalence Ratio; CI, Confidence Interval*.

a *p-value of Wald's test*.

## Discussion

Sarcopenia is a generalized and progressive disorder of skeletal muscle mass and is related to increased risk of adverse outcomes such as falls, fractures, physical frailty, and mortality in CKD patients (6, 23, 24). However, few studies had investigated the impact of PhA, Inflammatory Cytokines and ARBs-blockers, and sarcopenia prevalence in ND-CKD patients.

In our 139 ND-CKD patients, we described a prevalence of sarcopenia of 20.9%, using APMMI, assessed by BIA, and *HGS* to assess muscular strength, as recommended for EWGSOP2. Pereira et al., evaluating 287 ND-CKD patients, and using EWGSOP1 criteria, has described a sarcopenia prevalence of 9.8%, and 5.9% by assessing muscle mass index by anthropometric and BIA parameters, respectively (2). Otherwise, De Souza et al., analyzing 100 ND-CKD patients for sarcopenia, has described a 11.9%, and 28.7% defined by EWGSOP1 and FNIH criteria, respectively (3), while Zhou et al., described a sarcopenia prevalence of 14% by using APMMI accessed by Dual Energy X-Ray Absorption Densitometer (DEXA), as recommended for EWGSOP1 (25). Vettoretti et al., have found a similar value of sarcopenia prevalence (24%), compared to our study. Authors applied BIA analysis associated with anthropometric measurement to assess the muscle mass of the CKD patients evaluated (7).

As it is shown, sarcopenia prevalence varies according to the authors (2, 3, 25) and this finding can be explained partially by the different cut-offs of APMMI and HGS, defined by the guidelines adopted (16). Nonetheless, there is not a specific sarcopenia guideline for the ND-CKD population. In this way, studies have reported lower sarcopenia predominance when FNIH criteria is applied when compared to EWGSOP (26).

In our evaluated patients, sarcopenia diagnosis was performed using EWGSOP2 criteria, and body composition of our ND-CKD patients was evaluated by an octopolar BIA device that uses a 4-body compartment model (20), wich describes more accurately body compartments and also muscle mass than tetrapolar devices, commonly used in most clinical trials and assess muscle mass more accurately than tetrapolar devices commonly used in most clinical trials.

Regarding sarcopenia severity, characterized by decreased muscle performance and assessed by *GS*, only 2.9% of our ND-CKD population were classified as having severe sarcopenia. Dos Reis et al. also reported a low prevalence of severe sarcopenia of 5.7% in 129 kidney transplanted patients (27). Furthermore, in our study, the sarcopenic group had the lowest medians on *GS* when compared to non-sarcopenic patients and this finding was statistically significant in bivariate analysis (median = 1.00 points; *p*-value < 0.001). Similar results were found by De Souza et al. who evaluated 100 ND-CKD patients and found a lower GS in sarcopenic individuals (3).

Considering the association between eGFR and CKD stage with sarcopenia, our study shows a higher prevalence of sarcopenia in more advanced stages of CKD, however, no statistical difference was found. Moon et al., described a progressive increase of sarcopenia prevalence with the worsening in CKD stage in a population cohort of more than eleven thousand individuals (28). Surprisingly the authors had defined sarcopenia by applying a muscle index derived from appendicular muscle mass, assessed by DEXA. Likewise, Zhou et al., demonstrates that in ND-CKD patients G3-G5, loss of lean body mass (LBM) and APMMI and sarcopenia prevalence was significantly correlated with GFR decline (25). In addition, it becomes difficult to compare our results, derived from EWGSOP2 guidelines, with those described by previous studies, since they used different muscular parameters cut-offs for sarcopenia diagnosis as discussed above. Therefore, we can presume it's not clear the relationship between GFR decline and sarcopenia occurrence in ND-CKD patients based on current literature.

Moreover, in our cohort, individuals with sarcopenia had a lower BMI when compared to non-sarcopenic individuals. Along, in the multivariate model, BMI was not shown to be independently associated with sarcopenia in our ND-CKD patients. Other authors had demonstrated the association between lower BMI with sarcopenia in ND-CKD individuals (29). On the other hand, De Souza et al., reported that the highest BMI depicts an independent factor associated with sarcopenia in the ND-CKD assessed population and that can be explained by the low capacity of BMI to distinguish body composition (3). Furthermore, in our study, patients with sarcopenia presented lower PhA values when compared to non-sarcopenic individuals (4.5° vs. 5.6°). In multivariate analysis, after excluding the confounding variables, PhA was shown to be associated to sarcopenia. More than that, it was determined that a 1-unit increase in PhA was associated with a 63.6% decrease in PR for sarcopenia in our ND-CKD population.

PhA is a BIA-derived parameter that reflects body water distribution (30), and high values of PhA suggest increased cellularity, such as greater fat-free mass. In clinical practice, PhA is associated with strength (27), muscle mass (15, 17), clinical outcome, chronic inflammation and oxidative stress and nutritional status (15, 30) in patients with chronic diseases, such as CKD (31). Besides, PhA has been shown as a predictor of muscle function (14) and the recent EWGSOP2 guideline determined that it can be used as a marker of muscle quality for sarcopenia assessment (16).

Our results corroborate Kosoku et al. who demonstrate that both PhA and BMI were negatively correlated with sarcopenia in kidney transplant recipients (15). In the same way Han et al. described that, in stage G5 malnourished diabetic CKD patients, PhA < 4.17° was associated with eGFR, albumin levels, and LBM Index (17). Conversely, Dos Reis et al. did not find a correlation between PhA and sarcopenia in kidney transplant recipients (27). These different patterns of results, found by these authors, could be explained in part to the higher cut-off points for PhA adopted and from the lowest mean age of the individuals evaluated compared to our ND-CKD population.

Creatinine is another important marker used in clinical practice to evaluate renal function in the general population and in ND-CKD individuals (19). This molecule is a waste product of creatine metabolism, and its serum levels vary directly with muscle mass, animal protein intake, age, gender, use of some drugs, and presence of other chronic diseases (32). Our results demonstrated that sarcopenic ND-CKD patients presented lower creatinine levels when compared to non-sarcopenic, and these results were statistically significant in bivariate and multivariate analysis. Moreover, we also demonstrate that a one-unit increase in creatinine levels leads to a 21.2% reduction in PR for sarcopenia.

Since Patel et al. demonstrates a positive correlation between lean mass in maintenance hemodialysis patients, evaluated by DEXA or by an estimated equation using serum creatinine levels, other authors analyzed the association between serum creatinine with sarcopenia in CKD individuals (33). Lin et al. evaluated a strong relationship between the creatinine/cystatin C ratio, muscle mass and strength in 272 ND-CKD patients (34). In kidney transplant recipients, Yanishi et al. found a positive correlation between creatinine /cystatin C ratio and serum creatinine with muscle mass assessed by DEXA (35). In contrast, De Souza et al. and Dierkes et al. found no statistically significant connection between creatinine levels and sarcopenia in ND-CKD patients and in hemodialysis and kidney transplant recipients, respectively (3, 36).

Kidneys contribute about 20% of the endogenous synthesis of guanidinoacetic acid, a direct precursor of creatine production in the human body. The association of progressive loss of renal function and the low intake of animal protein in individuals with CKD could promote a negative balance of body creatine in these patients, which could favor the onset of sarcopenia, fatigue, loss of cognition, worsening in quality of life and increased mortality (37). In fact, the low levels of creatinine found in our sarcopenic ND-CKD patients could indirectly reflect a muscular creatine deficiency, consequence of low protein intake and decreased renal synthesis of this important muscle amino acid.

Systemic chronic inflammation and increased inflammatory markers such as CRP, IL-6, and TNF-α are related to increased muscle mass degradation and appearance of sarcopenia in ND-CKD patients (23, 24). Regarding the changes in muscle metabolism caused by IL-6, the mechanisms seem to be linked to the unbalance of muscle protein turnover with increased proteolysis, by activation of the ubiquitin-proteasome system, associated with blocking of protein synthesis, resulting from the blocking of signaling in the pathway of IGF-1-Akt/mammalian target of rapamycin (mTOR) caused by this cytokine (9).

In our study, IL-6 levels were associated with sarcopenia. Furthermore, it was established that the increase in 1 unit of this marker increases PR for sarcopenia by 6%. Conversely, De Souza et al. demonstrate that IL-4 levels, but not IL-6 or CRP, correlates with GS and lower limb muscle mass in ND-CKD patients (3). Besides, Vettoretti et al. found no relationship between CRP, TNF-α, IL-6, and other inflammatory markers with sarcopenia in ND-CKD individuals (7). Derived from IL-6 stimulus in the liver, CRP is a huge marker of inflammation and mortality in the CKD population (12). CRP levels' relationship to muscle wasting was already described in CKD patients and the general population (8). Our ND-CKD sarcopenic patients presented higher levels of CRP when compared to non-sarcopenic patients in bivariate analysis but not in a multivariate model. While in maintenance hemodialysis patients, it is reported that CRP levels positively correlate with synthesis, degradation, and negative protein balance in muscle (38), in the ND-CKD population, more research is needed to elucidate the role of inflammatory markers with sarcopenia in these individuals.

Elevation of AT2 in CKD is implicated in sarcopenia pathophysiology in this population (10). AT2 elevation promotes a blockade of IGF-1 receptors in muscle cells, triggering activation of intracellular pathways of caspase 3 and transforming growth factor-beta, increasing systemic inflammation and inhibition of muscle stem cells, which leads to increased muscle protein catabolism (11). Nonetheless, our results demonstrate that there was no correlation between the use of ARBs or ACEI drugs and sarcopenia in our ND-CKD population. In the same way, Ishikawa et al. evaluated 260 ND-CKD patients and demonstrated that loop diuretics but not ARBs significantly associate with sarcopenia in those individuals (29). Otherwise, Lin et al. demonstrated that ARBs have a protective effect on muscle strength loss in 160 maintenance hemodialysis patients. These divergent data indicate that more research should be performed to highlight the role of RAAS blockers in ND-CKD patients (39).

In our study, we used the most recent criteria of EWGSOP2, not only for sarcopenia diagnosis and prevalence but also to classify patients according to sarcopenia severity, based on muscle performance parameters. Besides, we assessed muscle mass using an octopolar BIA device, an easy to handle and available equipment for use in daily clinical practice. We also investigated the main factor that could correlate to sarcopenia in ND-CKD and evaluated the role of RAAS blockers drugs, PhA, and inflammation on its occurrence. Our multivariate model, demonstrate that PhA, in association with creatinine, a common renal function, and nutritional marker, and IL-6, a chronic inflammation marker, have, all together, a robust association to sarcopenia in ND-CKD patients. To the best of our knowledge, this is the first study that evaluated the association of PhA with sarcopenia and its components in stage 3 to 5 ND-CKD individuals. Even so, our findings did not support the use of RAAS blockers drugs on managing uremic sarcopenia in the ND-CKD patients.

However, limited by the cross-sectional characteristic of our study, we cannot infer a causal relationship between the multivariate model variables and sarcopenia in our ND-CKD patients. Along with that, the lack of a uniform criteria for sarcopenia diagnosis, which makes difficult, not only its recognition, but also the comparison between the different research on CKD population.

In this way, we suggest that further studies should be performed to better understand the role of PhA, IL-6 and creatinine levels in the diagnosis and management of sarcopenia in the ND-CKD population. Moreover, a uniform criterion must be urgently defined for a correct sarcopenia diagnosis in these individuals.

## Data Availability Statement

The raw data supporting the conclusions of this article will be made available by the authors, without undue reservation.

## Ethics Statement

The studies involving human participants were reviewed and approved by Hospital das Clínicas of the Federal University of Pernambuco (HC-UFPE) Research Ethics Committee/Number 3.258.943. The patients/participants provided their written informed consent to participate in this study.

## Author Contributions

GA, PC, and FS: study design, data analysis, and approving final version of manuscript. GA: study conduct. GA, CC, BS, and RA: data collection. GA: data interpretation and drafting manuscript. JL-F: infrastructure support. GV, LV, GA, JL-F, TF, MS, and AC: revising manuscript. All authors contributed to the article and approved the submitted version.

## Funding

This work was supported by the National Council for Scientific and Technological Development, through the Project Numbers 427243/2016-5 and 470702/2014-1. The funding entity had no role in study design, data collection and analysis, or decision to publish.

## Conflict of Interest

The authors declare that the research was conducted in the absence of any commercial or financial relationships that could be construed as a potential conflict of interest.

## Publisher's Note

All claims expressed in this article are solely those of the authors and do not necessarily represent those of their affiliated organizations, or those of the publisher, the editors and the reviewers. Any product that may be evaluated in this article, or claim that may be made by its manufacturer, is not guaranteed or endorsed by the publisher.
